# Fractionated radiation suppresses Kruppel-like factor 2 pathway to a greater extent than by single exposure to the same total dose

**DOI:** 10.1038/s41598-020-64672-3

**Published:** 2020-05-07

**Authors:** Ratan Sadhukhan, Justin W. C. Leung, Sarthak Garg, Kimberly J. Krager, Alena V. Savenka, Alexei G. Basnakian, Rupak Pathak

**Affiliations:** 1 Division of Radiation Health, Department of Pharmaceutical Sciences, College of Pharmacy, University of Arkansas for Medical Sciences, Little Rock, AR USA; 2 Department of Radiation Oncology, College of Medicine, University of Arkansas for Medical Sciences, Little Rock, AR USA; 3 Department of Pharmacology and Toxicology, University of Arkansas for Medical Sciences, Little Rock, AR USA; 4 Central Arkansas Veterans Healthcare System, Little Rock, AR USA

**Keywords:** Stress signalling, Transcriptional regulatory elements

## Abstract

Kruppel-like factor 2 (KLF2) is a positive transcriptional regulator of several endothelial protective molecules, including thrombomodulin (TM), a surface receptor, and endothelial nitric oxide synthase (eNOS), an enzyme that generates nitric oxide (NO). Loss of TM and eNOS causes endothelial dysfunction, which results in suppressed generation of activated protein C (APC) by TM-thrombin complex and in upregulation of intercellular adhesion molecule 1 (ICAM-1). Mechanistic studies revealed that activation of extracellular signal-regulated kinase 5 (ERK5) via upregulation of myocyte enhancer factor 2 (MEF2) induces KLF2 expression. Radiation causes endothelial dysfunction, but no study has investigated radiation’s effects on the KLF2 pathway. Because fractionated radiation is routinely used during cancer radiotherapy, we decided to delineate the effects of radiation dose fractionation on the KLF2 signaling cascade at early time points (up to 24 h). We exposed human primary endothelial cells to radiation as a series of fractionated or as a single exposure, with the same total dose delivered to each group. We measured the expression and activity of critical members of the KLF2 pathway at subsequent time points, and determined whether pharmacological upregulation of KLF2 can reverse the radiation effects. Compared to single exposure, fractionated radiation profoundly suppressed KLF2, TM, and eNOS levels, subdued APC generation, declined KLF2 binding ability to TM and eNOS promoters, enhanced ICAM-1 expression, and decreased expression of upstream regulators of KLF2 (ERK5 and MEF2). Pharmacological inhibitors of the mevalonate pathway prevented fractionated-radiation–induced suppression of KLF2, TM, and eNOS expression. Finally, fractionated irradiation to thoracic region more profoundly suppressed KLF2 and enhanced ICAM-1 expression than single exposure in the lung at 24 h. These data clearly indicate that radiation dose fractionation plays a critical role in modulating levels of KLF2, its upstream regulators, and its downstream target molecules in endothelial cells. Our findings will provide important insights for selecting fractionated regimens during radiotherapy and for developing strategies to alleviate radiotherapy-induced toxicity to healthy tissues.

## Introduction

Endothelium—the inner lining of blood and lymphatic vessels―is composed of endothelial cells and forms a critical component of all the body’s organs. Under normal physiological conditions, endothelium acts as a barrier between blood and surrounding tissues, maintains vascular tone, regulates blood homeostasis, provides an anticoagulant surface, and restricts proliferation of vascular smooth muscle cells^1^. Mechanistic studies revealed that all these endothelial-mediated beneficial functions are primarily exerted by thrombomodulin (TM), an endothelial surface receptor, and endothelial nitric oxide synthase (eNOS), an enzyme that generates nitric oxide (NO)^2–6^. TM has intrinsic antioxidant and anti-inflammatory properties^2,4^, and TM-thrombin complex enhances generation of activated protein C (APC), which exerts further anti-inflammatory, anti-coagulant, and anti-oxidant effects^2,7^. On the other hand, eNOS-derived NO facilitates vasodilation, inhibits platelet aggregation, and prevents platelets and leukocytes from adhering to the endothelium, thus limiting inflammation^6^.

Loss of TM and eNOS are the “hallmark” features of endothelial dysfunction^3,4,8^. A characteristic feature of endothelial dysfunction is upregulated cell-surface levels of adhesion molecules, such as intercellular adhesion molecule 1 (ICAM-1), vascular cell adhesion molecule 1 (VCAM-1), and E-selectin^9^. These adhesion molecules facilitate interaction and transendothelial migration of hematopoietic immune cells^10^. Adherence of immune cells to the endothelium surface and their subsequent migration to subendothelial tissues triggers inflammation by activating various pathways, including NF-κB^11,12^. Dysfunctional endothelium results in loss of barrier integrity and impaired blood flow, which causes tissue edema, ischemia, and hypoxia that subsequently trigger other pathological cascades, including inflammation, oxidative stress, excessive thrombosis, and fibrotic changes^8,13,14^. All these adverse events increase the risk of developing cardiovascular, pulmonary, neurological, and gastrointestinal disease^15,16^.

We and others demonstrated that exposure to a single dose of ionizing radiation (IR) suppresses expression and functioning of TM^17^ and eNOS^18^, resulting in endothelial dysfunction, and that administration of recombinant TM^19^ or treatment with pharmacological inducers of TM and eNOS mitigate the damage caused by single exposure of IR^18,20,21^. These data clearly indicate that TM and eNOS play critical roles in modifying the biological response to a single exposure of IR. Clinical studies showed that damage to TM and eNOS may occur during or after fractionated radiotherapy for cancer^22,23^, and a preclinical study demonstrated that localized fractionated radiation can suppress expression of TM in intestinal tissues^24^. However, these clinical and preclinical studies did not demonstrate whether the damage to these molecules is an exclusive effect of fractionated radiation on the endothelial cells, and they did not provide a mechanistic basis for the observed effects.

Both TM and eNOS are under positive transcriptional control of Kruppel like factor-2 (KLF2)^25–28^. KLF2 is highly expressed in lung tissue and is essential for normal lung development^29^. KLF2 suppression aggravates, while its upregulation mitigates, endothelial damage in response to pro-inflammatory cytokines^28,30,31^. In addition, transgenic mice that overexpress KLF2 develop less injury from ischemic stroke than mice deficient of *KLF2* gene^32^. It has been shown that KLF4, another member of the same family, has similar positive regulatory effects on TM and eNOS^33^. However, it is not known whether radiation (fractionated or single exposure) affects KLF2, KLF4, or their upstream regulators.

Extracellular signal-regulated kinase 5 (ERK5) is a critical upstream regulator of KLF2 in endothelial cells^34,35^. Activation of ERK5 leads to upregulation of myocyte enhancer factor 2 (MEF2), a known positive transcriptional regulator of KLF2^35^. Notably, statins, which are commonly used drugs for lowering lipids in circulation, inhibit a rate-limiting enzyme of the mevalonate pathway (3-hydroxy-3-methyl-glutaryl-coenzyme A reductase, HMGCR) and can upregulate KLF2 expression via activating ERK5 in an MEF2-dependent manner^36^. Other mevalonate pathway inhibitors, such as vitamin E family member gamma tocotrienol (GT3; inhibits HMGCR) and GGTI-298 (inhibits geranylgeranyltransferase I) also upregulate KLF2 expression in endothelial cells in the presence or absence of statins^37,38^. However, it is not known whether these mevalonate pathway inhibitors can modify the radiation effects on KLF2.

Here, we present results demonstrating that fractionated radiation suppressed the KLF2 pathway to a greater extent than a single acute exposure of the same total dose at early time points. Further, pharmacological inhibitors of the mevalonate pathway prevented these adverse changes in primary human endothelial cells.

## Materials and Methods

### Cell culture, reagents, and chemicals

Primary human umbilical vein endothelial cells (HUVECs) were obtained from American Type Culture Collection (ATCC; Manassas, VA, USA) and grown in vascular cell basal media supplemented with endothelial growth factors (ATCC). Cells were maintained with standard aseptic techniques in a humidified incubator with 5% CO_2_ at 37 °C and passaged every 2 to 3 days with a brief trypsin (Gibco; Grand Island, NY, USA) treatment. All the experiments were performed with cells between passage numbers 3 to 7 to avoid induction of endothelial cell senescence.

We purchased atorvastatin from Sigma-Aldrich (St. Louis, MO, USA), GT3 from Yasoo Health Inc. (Johnson City, TN, USA), and GGTI-298 from Tocris Bioscience (Minneapolis, MN, USA). Human protein C, thrombin, I-2581 (thrombin inhibitor), and Chromogenix S-2366 were from DiaPharma (West Chester, OH, USA). Bovine serum albumin (BSA) was obtained from Sigma. Vectashield antifade mounting media containing 4′,6-diamidino-2-phenylindole (DAPI) was purchased from Vector Laboratories (Burlingame, CA, USA).

### Cell irradiation

Cells were grown in T25 flasks (Corning, Corning, NY, USA) or 6-well plates (Corning) and were exposed to IR with a Shepherd Mark I ^137^Cs irradiator (model 25; J. L. Shepherd & Associates, San Fernando, CA, USA). We subjected cells to different regimens of fractionated radiation (3 or 5 fractions of 2 Gy and 2.5 Gy at 24-h inter-fraction intervals) and also various single-exposure doses (2, 5, 6, 7.5, 10, and 12.5 Gy). Sham irradiated (0 Gy) cells served as a control. In comparison studies, cells undergoing a single exposure were irradiated at the time when the last fraction was delivered to groups receiving fractionated radiation. For all groups (non-irradiated, single dose, and fractionated dose), cells were seeded at the same time, and culture medium was replaced every 24 h (just before radiation exposure) for 3 or 5 consecutive days. We replaced medium every 24 h to avoid accumulation of cell-derived toxic metabolites that may cause pH changes in the medium and contribute to cell death. To maintain identical experimental conditions, nonirradiated and single-exposure groups were transported to the radiation room along with the fractionated radiation group. Flasks or culture plates were placed on a turntable rotating at 6 rpm to ensure uniform distribution of the radiation dose. The average dose rate was 1.01 Gy/min and was corrected for decay each day. The fractionated radiation doses were chosen based on the clinical dose for conventional fractionated (2 Gy/fraction) and hypo-fractionated (2.5 Gy/fraction) radiotherapy to treat patients with cancer.

### Mouse irradiation, tissue procurement, and immunohistochemistry

All animal studies were carried out in strict accordance with the recommendations in the Guide for the Care and Use of Laboratory Animals of the National Institutes of Health. The animal protocol was approved by the Institutional Animal Care and Use Committee of the University of Arkansas for Medical Sciences (UAMS). Male C57BL/6 mice were obtained from The Jackson Laboratories (Bar Harbor, ME, USA); mice between 8 and 12 weeks of age were used for experiments. They were housed in conventional cages in a pathogen-free environment with controlled humidity, temperature, and a 12–12 light-dark cycle with free access to drinking water and standard chow (Teklad, Madison, WI, USA).

For lung irradiation, the animals were anethesized by isoflurane inhalation. Thoracic region was exposed to either 5 fractions of 4 Gy at 24 h intervals or a single dose of 20 Gy using an image guided Small Animal Radiation Research Platform or SARRP (Xstrahl, Suwanee, GA, USA). During irradiation single mouse was placed on a platform connected to the irradiator, and a cone beam-CT scan was performed using 60-kV and 0.4-mA photons filtered with aluminum (1 mm) to visualize the lung before delivering radiation. The software SARRP control and Muriplan were used to precisely target lungs and irradiation doses. The system uses a dual-focus, 0.15-mm Cu filtration, constant voltage X-ray source operating up to 225 kVp, which is mounted on a rotating gantry. All radiation experiments were performed in the morning to minimize possible diurnal effects.

For tissue harvest, mice were anesthetized with ketamine (100 mg/kg body weight) and xylazine (5 mg/kg body weight) solution administered intraperitoneally. Samples were collected 24 h after last fraction or single exposure and fixed in methanol-Carnoy’s solution for immunohistochemical studies or snap-frozen for molecular analysis. Immunohistochemical staining was performed with standard techniques using avidin-biotin complex, diaminobenzidine chromogen, and hematoxylin counterstaining. Appropriate positive and negative controls were included. Immunohistochemical staining for KLF2 and ICAM-1 was performed as described elsewhere^39^. Briefly, tissue sections were incubated with rabbit anti-KLF2 (1:100, LSBio, Seattle, WA, USA) or rabbit anti-ICAM-1 (1:250, Novus Biologicals, Centennial, CO, USA) antibodies for 2 hours. This was followed by a 30-min incubation with biotinylated goat anti-rabbit IgG at a 1:400 dilution (Vector Laboratories). Tissue sections were further incubated with avidin-biotin-peroxidase complex for 45 min (1:100, Vector Laboratories). Peroxidase binding was visualized with 0.5 mg/ml 3,3-diaminobenzidine (DAB) tetrahydrochloride solution (Sigma-Aldrich) and 0.003% H_2_O_2_ in TBS. Immunoreactivity was quantified using IHC profiler plug-in in ImageJ. Histogram data were then plotted to get the pixel intensity of DAB at the area of vascular endothelium.

### Western blot analysis

At different post-irradiation times (with or without treatment with mevalonate pathway inhibitors), cells were harvested, washed twice with ice-cold PBS, and lysed with RIPA lysis buffer (Boston BioProducts, Ashland, MA, USA) containing 1× protease and phosphatase inhibitors (ThermoFisher Scientific, Waltham, MA, USA). Cell lysates were sonicated (20 kHz) for 5 s on ice, incubated on ice for 5 min, and centrifuged at 10,000 *g* for 5 min at 4 °C; clear supernatants were transferred to clean tubes. Protein concentrations were determined with Pierce BCA protein assay kit (ThermoFisher Scientific). Equal amount of proteins (15–25 μg) were separated by SDS-PAGE (BioRad Laboratories, Hercules, CA, USA) and transferred onto 0.22-μm PVDF membranes (GE Healthcare; Chicago, IL, USA). The membranes were blocked with 5% nonfat dry milk in TBS-Tween-20 (TBS-T; Santa Cruz Biotechnology, Inc., Dallas, TX, USA) for 2 h at room temperature and probed with primary antibodies overnight at 4 °C. Membranes then were washed four times with TBS-T and incubated with appropriate horseradish peroxidase (HRP)-conjugated secondary antibodies (Cell Signaling Technology, Beverly, MA, USA) for 1 h at room temperature. Membranes were washed five times with TBS-T on a shaker. Protein bands were detected with enhanced chemiluminescence (ThermoFisher Scientific) and were visualized by exposing autoradiography film (Denville Scientific Inc., Metuchen, NJ, USA), which was densitometrically analyzed with ImageJ application to quantify protein levels.

### Immunofluorescence assay

Cells were grown on alcohol-sterilized clean cover glasses placed in the wells of 12-well culture plates. At various predetermined time points, cells were washed twice with ice-cold PBS and fixed with 4% paraformaldehyde (Fisher Scientific) in PBS for 10 min at 4 °C. After fixation, cells were washed with PBS and incubated with 0.25% Triton X-100 (Fisher Scientific) in PBS for 10 mins to permeabilize the cells. Permeabilized cells were blocked with 1% BSA/0.3 M glycine in PBS-T (PBS + 0.1% Tween 20) for 1 h. Next, cells were incubated overnight at 4 °C with primary antibody in 1% BSA (Sigma-Aldrich) containing PBS-T. Cells then were washed four times with PBS-T and incubated with fluorophore-conjugated secondary antibody for 1 h. Cells were washed six times to remove unbound secondary antibody and then mounted on a glass slide with DAPI-containing mounting medium (Vector Laboratories). Images were captured with a fluorescence microscope with the appropriate filter.

### Chromatin immunoprecipitation (ChIP) assay

The ChIP assay was performed as described elsewhere^27^ using Thermo Scientific Pierce Agarose ChIP Kit (Thermo Scientific). All ChIP experiments were performed in duplicate. Briefly, DNA associated with the specific immunoprecipitate using KLF2 antibody (Aviva Systems Biology, San Diego, CA, USA) or negative control using normal rabbit IgG was isolated and used as a template for PCR reaction, amplifying a 436-bp region of the TM and eNOS promoters with the putative Klf2 binding site or the *GAPDH* gene. A 153-bp fragment of the TM promoter between −239 and −86 was amplified with forward (5′AGA TGA AAG AGG GCT GCA CG3′) and reverse (5′ACC TCG CCG GGA TGA GTA AA3′) primers. A 237-bp region of eNOS promoter between −597 and −360 were amplified by PCR was amplified using forward (5′ACA GAG GAG TCA TCC TGC GA3′) and reverse (5′GTC TGT GGG CGT AAC ATC CC3′) primers. A 306-bp of the *GAPDH* gene was amplified using the primer provided by manufacturer (Thermo Scientific) and used as control. The cycling conditions for all three regions were 5 min at 95 °C, followed by 32 cycles of 30 sec at 95 °C, 30 sec at 60 °C and 45 sec at 72 °C. The PCR products were analyzed by 1.5% agarose gel electrophoresis.

### Primary and secondary antibodies

Antibodies to KLF2 (Cat # ab203591), KLF4 (Cat # ab106629), and MEF2 (Cat # ab64644) were purchased from Abcam (Burlingame, CA, USA). Antibodies to TM (Cat # NBP1-95319), ICAM-1 (Cat # NBP2-22541), eNOS (Cat # NB300–500), E-selectin (Cat #NBP1-45545), and gamma H2AX (Cat # NB100-384) were procured from Novus Biologicals. Antibodies to ERK5 (Cat #3372), phosphoERK5 (Cat #3371), and β-actin (Cat #4970) were obtained from Cell Signaling Technology Inc. (Beverly, MA, USA).

Anti-rabbit IgG-HRP (Cat #7074) and anti-mouse IgG-HRP (Cat #7076) secondary antibodies were purchased from Cell Signaling Technology Inc. Fluorochrome-conjugated goat anti-rabbit IgG (H + L)-Alexa Fluor 488 (Cat # A11034), donkey anti-goat IgG (H + L)-Alexa Fluor 568 (Cat # A11057), and goat anti-mouse IgG (H + L)-Alexa Fluor 488 (Cat # A11029) were procured from Invitrogen (ThermoFisher Scientific).

### Flow cytometry analysis to determine ICAM-1 protein level

Sham-irradiated cells and cells exposed to either 5 fractions or a single exposure of IR were harvested with a brief trypsin (0.025%) treatment, followed by washing with ice cold PBS. Cells were blocked with PBS containing 4% Fetal Bovine Serum for 30 min and incubated with ICAM-1 antibody in 100 μl PBS overnight at 4 °C. Cells were washed two times with PBS and incubated with secondary antibody conjugated with Alexa-488 for 1 h at room temperature. Data were acquired with an LSRII (BD Biosciences, San Jose, CA, USA) and analyzed with FlowJo Cytometry software from Tree Star Inc. (Ashland, OR, USA). In each treatment group, 10^4^ viable cells were analyzed.

### APC generation assay

APC assays were performed as described elsewhere^25^. Cells (2 × 10^4^ cells/well) were seeded in 96-well plates and exposed to various doses of IR. Twenty-four h after completion of radiation exposure, cells were washed with PBS and incubated with 60 μl reaction buffer (20 mM Tri-HCL, pH 7.4; 100 mM NaCl; 2.5 mM CaCl_2_; 0.5% BSA) containing thrombin (1 nM) and protein C (0.5 μM) for 1 h at 37 °C. After 1 h incubation, thrombin inhibitor was added to neutralize thrombin. After 10 min, 100 μl of chromogenic substrate S-2366 (0.5 mM) was added, and the kinetic change in absorbance at 450 nm was measured with a microplate reader (BioTek, Winooski, VT, USA).

### ELISA assay of NF-κB activation

Activation of NF-κB in nuclear extracts of HUVECs was measured with DNA-binding ELISA kits that measure the activity of NF-κB family members, such as p50, p52, p65, c-Rel, and RelB (Cat #43296; Active Motif, Carlsbad, CA, USA). Briefly, assays used equal amounts (5 μg) of nuclear extracts that were normalized for total protein concentration. Extracts were incubated with herring sperm DNA in binding buffer for 1 h at 37 °C with mild agitation. After incubation, the ELISA plate was washed 3 times with wash buffer, incubated with primary antibody for 1 h, washed again wash buffer and incubated with HRP-conjugated secondary antibody for 1 h. Unbound secondary antibody was washed away, and bound secondary antibody was detected by colorimetric reaction after addition of HRP substrate (i.e., TMB); absorbance was measured at 450 nm.

### Statistical analysis

Results are expressed as mean ± standard error of the mean. Data were analyzed with Prism software (version 4.0; GraphPad, San Diego, CA, USA). Differences in measured variables of experimental and control groups were assessed with *t* test; *p* < 0.05 was considered statistically significant.

## Results

### KLF2 is suppressed at a higher level by fractionated radiation than by a single exposure to the same total radiation dose

**Figure 1:**
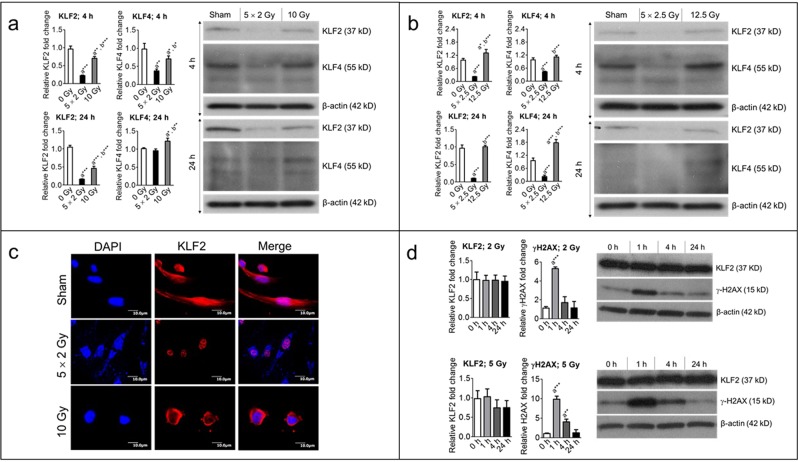
Fractionated, compared to single exposure, radiation profoundly suppresses levels of KLF2 and KLF4. Representative (3–5 independent experiments) Western blot analysis and quantification of KLF2 (n = 5) and KLF4 (n = 3) levels in whole-cell lysates from nonirradiated (sham) and irradiated HUVECs 4 h and 24 h after exposure to (**a**) either five fractions of 2 Gy (5 × 2 Gy) or single exposure to 10 Gy and (**b**) either five fractions of 2.5 Gy (5 × 2.5 Gy) or single exposure to 12.5 Gy. Fractions delivered at 24-h intervals. β-actin served as a loading control. **c** Representative photomicrograph (20× magnification) showing immunofluorescence of KLF2 (red) in nonirradiated (sham) and irradiated HUVECs 4 h after exposure to either five fractions of 2 Gy or single exposure to 10 Gy (n = 3). Nuclei were stained with DAPI (blue). **d** Representative Western blot analysis and quantification of KLF2 (n = 3) and γ-H2AX (n = 3) phosphorylation levels in nonirradiated (0 h) and irradiated HUVECs at indicated time intervals after single exposure to 2 Gy and 5 Gy. β-actin served as a loading control. (n, number of independent experiments performed; a, significant statistical difference between nonirradiated and irradiated groups; b, significant statistical difference between fractionated irradiation and single exposure; *, p < 0.05; **, p < 0.01; ***, p < 0.001).

We first determined the effects of radiation dose fractionation on KLF2 expression in primary HUVECs. For the fractionation group, cells were exposed to five fractions of 2 Gy or 2.5 Gy, with 24 h between each fraction. For the single-exposure group, cells were exposed to one dose of either 10 Gy or 12.5 Gy (the total doses delivered to the fraction group); the control group was exposed to sham irradiation. KLF2 expression 4 h and 24 h after irradiation was determined with Western blot analysis. At both time points and both doses, KLF2 expression was substantially suppressed in cells exposed to five fractions than in those with a single exposure (Fig. 1a,b), as compared to the control. For example, 4 h post-irradiation, five fractions of 2 Gy resulted in about 77% suppression, while a single dose of 10 Gy resulted in 25% suppression; similar results were observed 24 h post-irradiation (Fig. 1a). Interestingly, five fractions of 2.5 Gy caused significant decreases in KLF2 levels 4 h (about 80% decrease) and 24 h (approximately 90% decrease) post-irradiation, but a single 12.5-Gy exposure did not suppress KLF2 levels at either time point (Fig. 1b). We also used immunofluorescence to examine KLF2 expression and localization, and these results confirmed those of the Western blot analyses, indicating that KLF2 expression was more profoundly suppressed by fractionated radiation than by a single exposure (Fig. 1c). However, we observed greater nuclear localization of KLF2 after fractionated radiation than single exposure to same total dose at 24 h (Fig. 1c).

To investigate the effects of a lower number of fractions, we exposed HUVECs to three fractions of 2 or 2.5 Gy or to a single exposure of 6 Gy or 7.5 Gy (the total doses delivered by fractionation). As with five fractions, three fractions of radiation suppressed KLF2 levels to a greater level than a single exposure of either dose (Supplementary Fig. 1). These data clearly indicate that radiation dose fractionation plays a critical role in altering KLF2 expression. Because KLF2 was suppressed substantially by five fractions compared to three fractions, we performed the rest of our experiments with five fractions.

KLF2 and KLF4 have overlapping functions in endothelial cells^40^, so we investigated effects of fractionated and single-exposure irradiation on expression of KLF4. KLF4 levels, like those of KLF2, were significantly suppressed after fractionated radiation than after a single exposure (Fig. 1a,b), as compared to the control. For example, 4 h post-irradiation, KLF4 levels decreased by about 60% in response to five fractions of 2 Gy, but they decreased by only 20% in response to a single dose of 10 Gy (Fig. 1a). However, 24 h post-irradiation, KLF4 levels reached those of the control group after five fractions of 2 Gy, and a single exposure of 10 Gy caused about 12% increase in KLF4 levels, relative to those of the control (Fig. 1a).

To examine the possibility of dose-dependent effects of a single exposure on KLF2 suppression, we exposed HUVECs to a single dose of 2 Gy or 5 Gy. KLF2 levels were measured 1 h, 4 h, and 24 h post-irradiation. KLF2 levels did not change after a single exposure to either dose, but γ-H2A.X phosphorylation (a positive marker for radiation damage) changed significantly at the early time point, as expected (Fig. 1d). These data indicate that a single exposure to 2 or 5 Gy did not suppress KLF2 expression within 24 h.

### Fractionated radiation is more effective than a single exposure in downregulating target molecules downstream of KLF2

**Figure 2:**
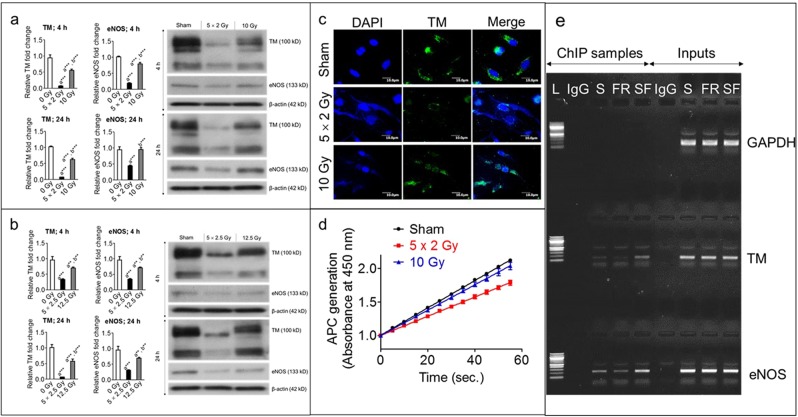
Fractionated, compared to single exposure, radiation more profoundly suppressed TM and eNOS. Representative (3 independent experiments) Western blot analysis and quantification of TM and eNOS levels in whole-cell lysates from nonirradiated (sham) and irradiated HUVECs 4 h and 24 h after exposure to (**a**) either five fractions of 2 Gy (5 × 2 Gy) or single exposure to 10 Gy and (**b**) either five fractions of 2.5 Gy (5 × 2.5 Gy) or single exposure to 12.5 Gy. Fractions were delivered at 24-h intervals. β-actin served as a loading control. **c** Representative photomicrograph (20× magnification) showing immunofluorescence of TM (green) in nonirradiated (sham) and irradiated HUVECs 4 h after exposure to either five fractions of 2 Gy or single exposure to 10 Gy (n = 3). Nuclei were stained with DAPI (blue). **d** APC generation in HUVECs 24 h after exposure to 0 Gy (sham), five fractions of 2 Gy, or single exposure to 10 Gy. Experiments were performed twice (n = 2) with four biological replicates. **e** ChIP assay showing KLF2’s binding ability to TM and eNOS promoter 24 h after 3 fractions of 2.5 Gy or single fraction of 7.5 Gy in HUVECs; Glyceraldehyde 3‐phosphate dehydrogenase (GAPDH) was used as control for ChIP assay. (L, ladder (100 bp); S, sham; FR, fractionated radiation; SF, single fraction).

To examine effects of radiation fractionation on KLF2’s downstream targets, we compared levels of TM and eNOS, after fractionated or single-exposure radiation. Western blot data demonstrated that TM expression was considerably decreased after fractionated radiation than after a single exposure to the same total doses (Fig. 2a,b). For example, five fractions of 2 Gy and 2.5 Gy caused, respectively, 90% and 70% suppression 4 h post-irradiation; at the same time point, single doses of 10 Gy and 12.5 Gy resulted in, respectively, 40% and 30% suppression. Similar trends in TM suppression were observed 24 h post-irradiation. We also measured TM expression with immunofluorescence and found that TM suppression was more pronounced after fractionated radiation than after single exposures (Fig. 2c). To investigate effects of fractionated and single-exposure radiation on TM activity, we measured APC generation in HUVECs after 24 h of five fractions of 2 Gy or a single dose of 10 Gy. APC generation was significantly lower after fractionated radiation than single exposure (Fig. 2d); similar effects on APC generation were observed after exposure to three fractions of 2 Gy or a single exposure to 6 Gy (Supplementary Fig. 2). These data clearly indicate that downregulation of TM expression and activity are more pronounced after fractionated radiation than after a single exposure.

Similarly, we measured eNOS protein levels at the same time points after exposure to identical radiation doses. As with TM, eNOS expression was suppressed to a greater extent after fractionated radiation than after a single exposure (Fig. 2a,b). For example, five fractions of 2 Gy and 2.5 Gy caused, respectively, 80% and 64% suppression of eNOS expression 4 h post-irradiation; at the same time point, single doses of 10 Gy and 12.5 Gy resulted in, respectively, 20% and 27% suppression. Similar trends were observed 24 h post-irradiation (Fig. 2a,b). These findings clearly suggest that suppression of KLF2 downstream targets is more pronounced after fractionated radiation than after a single exposure to the same total dose.

Because we observed fractionated radiation enhances nuclear localization of KLF2, which may increase the expression of downstream target molecules, we did perform ChIP assay to determine the binding ability of KLF2 to the promoters of TM and eNOS in HUVECs following fractionated radiation (3 fractions of 2.5 Gy at 24 h interval) and single exposure (7.5 Gy) to the same total dose. Our ChIP assay data revealed that binding ability of KLF2 to TM and eNOS promoter is substantially suppressed after fractionated radiation than single exposure (Fig. 2e).

### Expression of ICAM-1 is significantly increased after fractionated radiation than after a single exposure

**Figure 3:**
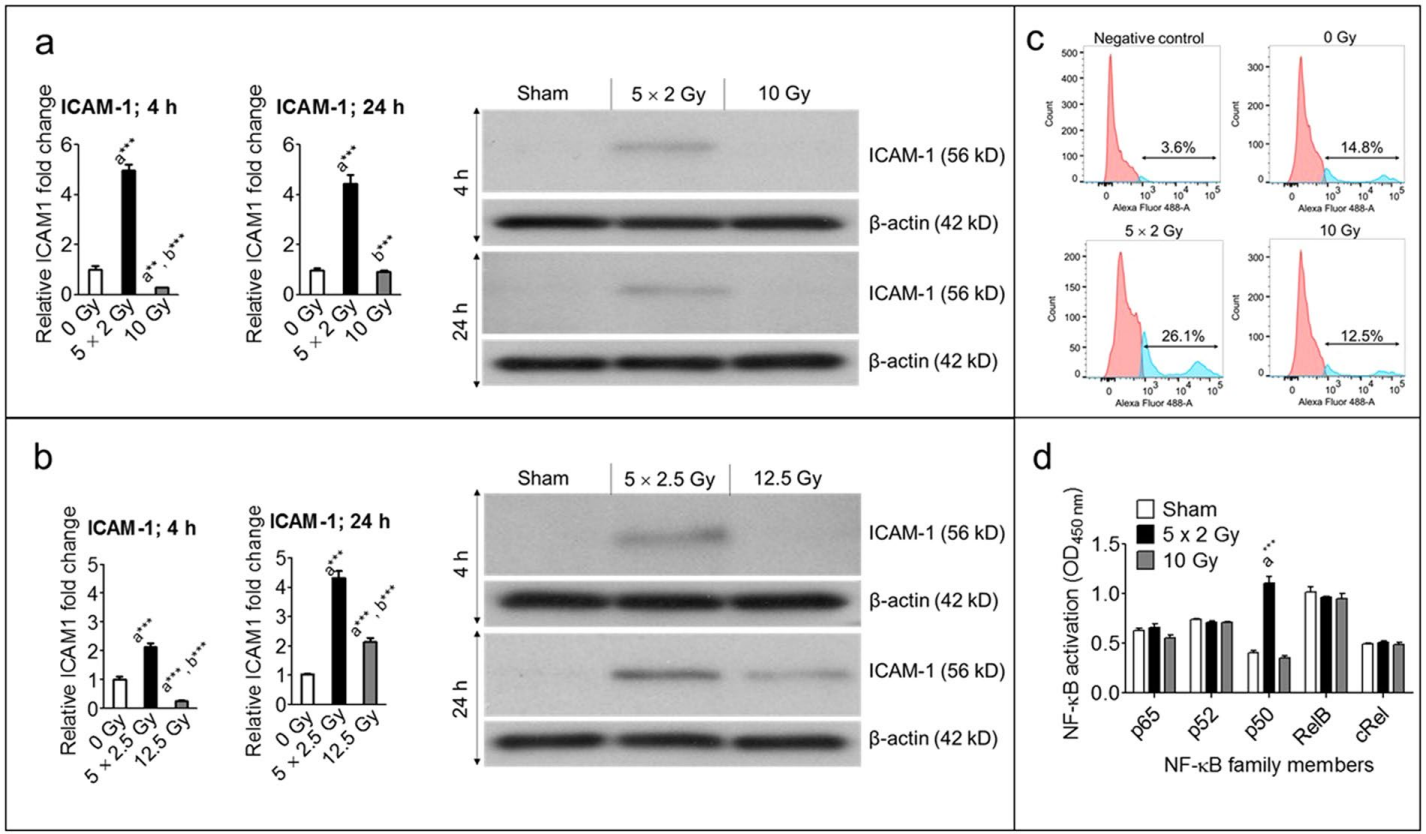
Fractionated, compared to single exposure, radiation more profoundly enhanced ICAM-1 expression. Representative (3 independent experiments) Western blot analysis and quantification of ICAM-1 levels in whole-cell lysates from nonirradiated (sham) and irradiated HUVECs 4 h and 24 h after exposure to (**a**) either five fractions of 2 Gy (5 × 2 Gy) or single exposure to 10 Gy and (**b**) either five fractions of 2.5 Gy (5 × 2.5 Gy) or single exposure to 12.5 Gy. Fractions delivered at 24-h intervals. β-actin served as a loading control. **c** Ectopic expression of ICAM-1 after exposure to 0 Gy, five fractions of 2 Gy, or 10 Gy, as measured by flow cytometry (n = 3). **d** NF-κB activation after 4 h of exposure to 0 Gy, five fractions of 2 Gy, or 10 Gy (n = 2).

In dysfunctional endothelium, expression of adhesion molecules on the surface (e.g., ICAM-1) are upregulated, which facilitates trapping of hematopoietic immune cells^9^. We measured, with Western blot analyses, expression of ICAM-1 after fractionated and single-exposure radiation at different time intervals. A significant increase in ICAM-1 expression was observed after fractionated radiation, but not after single exposure (Fig. 3a,b). For example, five fractions of 2 Gy and 2.5 Gy upregulated ICAM-1, respectively, 500% and 200% 4 h post-irradiation; at the same time point, single exposures of 10 Gy and 12.5 Gy each caused about 70% suppression of ICAM-1. In addition, we measured, with flow cytometry, ectopic expression of ICAM-1 and observed that ICAM-1 expression was induced to a greater extent after fractionated radiation than after a single exposure of the same total dose (Fig. 3c). However, two other endothelial cell adhesion molecules, E-selectin and VCAM-1, did not increase after fractionated radiation; rather, these molecules were significantly suppressed (Supplementary Fig. 3a,b). These data indicate that ICAM-1 upregulation after fractionated radiation may not be NF-κB-dependent. To investigate the role of the NF-κB pathway, after HUVECs were exposed to fractionated and single-exposure radiation, we extracted NF-κB family proteins (p65, p52, p50, RelB, and cRel) from their nuclei and measured the ability of the proteins to bind to the NF-κB consensus site (5′-GGGACTTTCC-3′). All proteins except p50, which has no transcriptional activity, bound to the consensus sequence at similar levels after fractionated radiation and after the single exposure, suggesting fractionated and single exposure exert similar effects on NF-κB transcriptional activity (Fig. 3d).

### Upstream regulators of KLF2 are suppressed to a greater extent after fractionated radiation than after a single exposure of the same total dose

**Figure 4:**
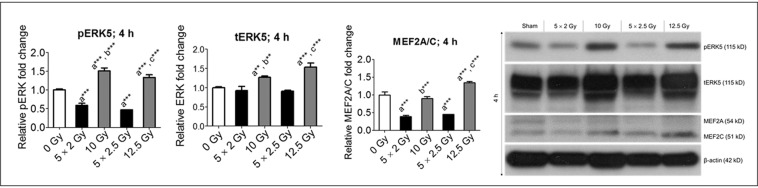
Fractionated, compared to single exposure, radiation more profoundly suppressed upstream regulators of KLF2. Representative Western blot analysis and quantification of pERK5, tERK5, and MEF2A/C levels in nonirradiated (0 Gy) and irradiated HUVECs 4 h after exposure to either five fractions of 2 Gy (5 × 2 Gy), five fractions of 2.5 Gy (5 × 2.5 Gy), single exposure to 10 Gy, or single exposure to 12.5 Gy (n = 3). β-actin served as a loading control. (n, number of independent experiments performed; a, significant statistical difference between nonirradiated and irradiated groups; b, significant statistical difference between five fractions of 2 Gy vs single exposure to 10 Gy; c, significant statistical difference between five fractions of 2.5 Gy vs single exposure to 12.5 Gy; *, p < 0.05; **, p < 0.01; ***, p < 0.001).

To elucidate the molecular mechanisms involved in mediating the effects of fractionated radiation on KLF2 suppression, we measured expression of ERK5 and MEF2, upstream regulators of KLF2. Phosphorylation of ERK5 (resulting in pERK5) positively regulates KLF2 expression^36^. Relative to the control, fractionated radiation significantly suppressed ERK5 phosphorylation, but single exposure to the same total dose increased ERK5 phosphorylation (Fig. 4). For example, 4 h post-irradiation, five fractions of 2 Gy and 2.5 Gy resulted in, respectively, about 35% and 50% suppression of pERK5; at the same time point, single exposures of 10 and 12.5 Gy resulted in, respectively, 50% and 40% increases in pERK5. We observed no change in total ERK5 (tERK5) after fractionated radiation, but tERK5 increased 30% and 55%, respectively, after single exposures of 10 Gy and 12.5 Gy. Because ERK5 regulates KLF2 via MEF2^35^, we also measured MEF2A/C expression. MEF2A/C was significantly suppressed after fractionated radiation (Fig. 4). For example, 4 h post-irradiation, MEF2A/C was suppressed by approximately 55% after exposure to five fractions of 2 and 2.5 Gy; at same time point, MEF2A/C was not suppressed after single exposures to 10 Gy and 12.5 Gy, and it increased by about 40% after a single exposure to 12.5 Gy. These data clearly indicate that fractionated radiation suppresses the ERK5-MEF2-KLF2A/C axis, but a single exposure does not.

### Mevalonate pathway inhibitors prevent fractionated radiation-induced suppression of KLF2 and its downstream targets

**Figure 5:**
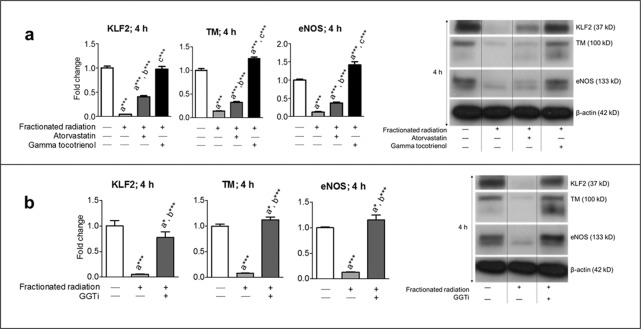
Mevalonate pathway inhibitors reversed fractionated-radiation–induced suppression of KLF2 and its downstream target molecules. Representative Western blot analysis and quantification of KLF2, TM, and eNOS 4 h after exposure to five fractions of 2 Gy (**a**) in presence or absence of atorvastatin (1 μM) or GT3 (5 μM) and (**b**) in presence or absence of GGTi (10 μM) (n = 3). β-actin served as a loading control. (n, number of independent experiments performed; a, significant statistical difference between nonirradiated and irradiated groups; b, significant statistical difference between fractionated irradiation and single exposure; *, p < 0.05; **, p < 0.01; ***, p < 0.001).

We tested the efficacy of inhibitors of the mevalonate pathway, specifically, HMG-CoA reductase inhibitors (i.e., atorvastatin and GT3) and GGTi, in attenuating KLF2 suppression after fractionated radiation. We and others have shown HMG-CoA reductase inhibitors enhanced the expression of KLF2 and its downstream target molecules, such as TM and eNOS and provided radiation protection^28,30,38,39,41^. Treatment with atorvastatin, GT3, and GGTi significantly prevented fractionated radiation-induced suppression of not only KLF2 but also its downstream target molecules TM and eNOS (Fig. 5). These data indicate that targeting the mevalonate pathway could be a novel therapeutic strategy to prevent endothelial injury induced by fractionated radiation.

### Lung KLF2 is suppressed and ICAM-1 is significantly enhanced by fractionated radiation than by a single exposure to the same total radiation dose

**Figure 6:**
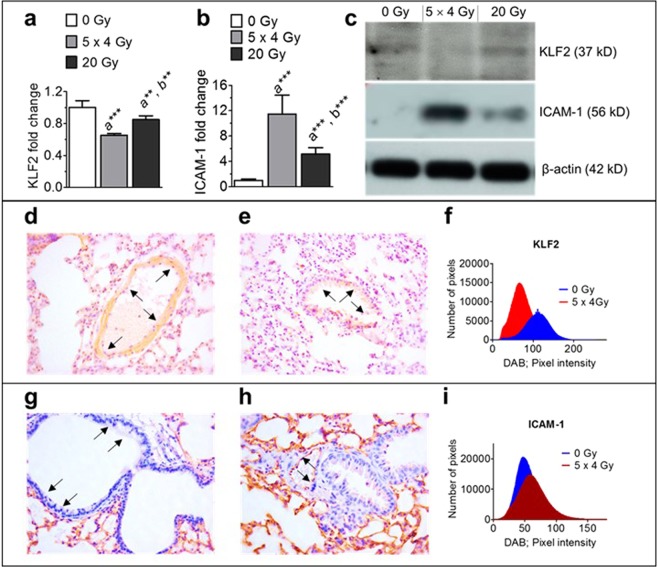
Fractionated thoracic irradiation suppressed KLF2 and enhanced ICAM-1 levels in the lung. Quantification of KLF2 (**a**) and ICAM-1 (**b**) protein levels and representative Western blot analysis (**c**) in the lung tissue of mice (n = 6) at 24 h following 5 fractions of 4 Gy at 24 h intervals or single exposure to 20 Gy. β-actin served as a loading control. KLF2 immunostaining in the lung tissue samples of sham irradiated (**d**), irradiated (**e**), and quantitation (**f**) at 24 h after exposure to 5 fractions of 4 Gy at 24 h intervals or single exposure to 20 Gy. ICAM-1 immunostaining in the lung tissue samples of sham irradiated (**g**), or irradiated (**h**), and quantitation (**i**) at 24 h after exposure to 5 fractions of 4 Gy at 24 h intervals or single exposure to 20 Gy. (n, number of independent experiments performed; a, significant statistical difference between nonirradiated and irradiated groups; b, significant statistical difference between fractionated irradiation and single exposure; *p < 0.05; **p < 0.01; ***p < 0.001).

Finally, we confirmed our key *in vitro* findings by *in vivo* studies after fractionated and single thoracic radiation exposure to the same total dose. We observed 24 h after exposure to 5 fractions of 4 Gy more profoundly suppressed KLF2 and enhanced ICAM-1 expression in the lung tissue of mice than single dose of 20 Gy as detected by Western blot analysis (Fig. 6a–c). In addition, less KLF2 (Fig. 6d–f) and more ICAM-1 (Fig. 6g–i) immunoreactivity were observed in the lung tissue after fractionated radiation as compared to sham irradiated groups.

## Discussion

According to an American Cancer Society Report, an estimated 1.7 million patients received a new cancer diagnosis in 2018; of these, 50–60% will receive fractionated radiotherapy. Unfortunately, tumor regression due to radiotherapy strongly correlates with healthy tissue toxicity, which often becomes the major dose-limiting factor^42^. The pathogenesis of radiotherapy-induced toxicity to healthy tissue is highly complex^43^. Notably, endothelial dysfunction is considered a critical determinant of early (within days), acute (within weeks), and delayed (in months or years) adverse effects of radiotherapy^22,44,45^. Therefore, understanding the mechanisms of endothelial dysfunction induced by fractionated radiation is critical for counteracting the side effects of radiotherapy.

TM and eNOS impairment cause endothelial dysfunction. Damage to TM^23^ and partial loss of eNOS-dependent vascular function^22^ were observed in patients undergoing fractionated radiotherapy for lung and breast cancer, respectively. Significantly fewer TM-positive vessels were observed in both normal and tumor tissues obtained from patients with rectal cancer who received adjuvant therapy either as 30 Gy in 10 fractions or as 45 Gy in 25 fractions with concomitant 5-fluorouracil treatment^46^. Moreover, in rat intestinal tissue, localized fractionated radiation suppresses TM expression^24^. All these studies suggest that endothelial dysfunction occurs after fractionated radiation.

Results of these *in vivo* studies, however, do not address whether fractionated-radiation–induced endothelial dysfunction is an exclusive effect of radiation on the endothelial cells or is a consequence of radiation-induced indirect adverse effects on the endothelial cells. Radiation may indirectly exert adverse effects on the endothelium by various ways, such as, by enhancing production of pro-inflammatory cytokines through immune cells, or by facilitating attachment of leukocytes and platelets on the activated endothelial cell surface, or by inducing sepsis. Our current studies demonstrated sole effects of fractionated radiation on primary human endothelial cells *in vitro*. We observed that, in comparison to a single exposure, fractionated radiation significantly suppressed levels of TM and eNOS and generation of APC in HUVECs. Therefore, understanding how fractionated radiation exerts more profound suppression of TM and eNOS than single exposure in endothelial cells may provide critical insights for strategies to prevent or mitigate endothelial damage induced by therapeutic radiation.

Mechanistic studies demonstrated that KLF2 is a positive transcriptional regulators of TM and eNOS^27,28,33^. Pro-inflammatory cytokines and bacterial lipopolysaccharide suppress KLF2 and its downstream target molecules, including TM and eNOS^28,30,33^. Therefore, we hypothesized that negative effects on expression of KLF2 that are exerted by fractionated radiation may be more profound than those exerted by a single exposure. Indeed, we observed that KLF2 was suppressed to a greater extent after fractionated radiation than after a single exposure of the same total dose in HUVECs. To eliminate the possibility of higher cell death after single acute exposure is responsible for removing HUVECs without downregulation of the KLF2 signaling pathway, we closely monitored the cell death by light microscopy after fractionated and acute exposures. No significant difference in cell death was observed between single acute exposure and fractionated radiation at 24 h (data not shown). Notably, we observed fractionated radiation results in more accumulation of KLF2 in the nucleus than single exposure. Enhanced nuclear KLF2 localization may enhance the expression of its downstream target molecules, including TM and eNOS. However, our ChIP assay data revealed that the binding ability of KLF2 in TM and eNOS promoter is significantly less than single exposure. In addition, fractionated thoracic irradiation causes more suppression of KLF2 in the lung tissue than single exposure to the same total dose at 24 h. These data clearly indicate that radiation dose fractionation played a critical role in altering expression of KLF2, sub-cellular localization, and interaction with the promoters of downstream target molecules. Importantly, fractionated radiation also suppressed KLF4 to a greater extent than single exposure. KLF2 and KLF4 play critical role in maintaining endothelial identity and integrity^40^, so our findings may be particularly relevant to various vascular disease states that may occur during and after radiotherapy, including atherosclerosis and aortic aneurysm.

A characteristic feature of vascular endothelial injury is upregulation of ICAM-1, a critical inflammatory mediator^9^; KLF2 suppression upregulates ICAM-1 expression^47^. Because we observed that levels of KLF2, TM, and eNOS were significant lower after fractionated radiation than after single exposure to the same total dose, which suggests more endothelial damage, we assessed potential alterations in expression of ICAM-1. We observed that ICAM-1 expression was significantly increased in HUVECs and lung tissue of mice after fractionated radiation than single exposure to the same total dose; others have similarly observed greater increases in cellular ICAM-1 expression after fractionated radiation, relative to single exposure^48^. The previous report also demonstrated that fractionated radiation-induced upregulation of ICAM-1 is NF-κB-dependent^48^, but we did not observe activation of the NF-κB pathway. This apparent discrepancy could be due to differences in fractionation regimens; we delivered five fractions of 2 and 2.5 Gy, while the other study used two fractions of 0.125 and 0.25 Gy. Notably, activation of the NF-κB pathway in endothelial cells also upregulates expression of VCAM-1 and E-selectin^49^; we did not detect upregulation of VCAM-1 or E-selectin, suggesting that ICAM-1 upregulation after fractionated radiation may not be NF-κB-dependent. Therefore, mitigation of radiation-induced endothelial damage may require understanding of signaling pathways involved in the ability of fractionated radiation not only to suppress KLF2 and its downstream target molecules, but also to upregulate ICAM-1.

Within the mitogen-activated protein kinase (MAPK)/ERK pathway, activation of MAPK/ERK protein-serine kinase 5 (MEK5) upregulates KLF2 and KLF4 in endothelial cells by phosphorylating Thr218 and Tyr220 in the activation loop of ERK5, which in turn upregulates MEF2, a positive transcriptional regulator of KLF2 and KLF4^34,36,50^. Pro-inflammatory cytokines downregulate KLF2 and KLF4 in endothelial cells by suppressing the MEK5/ERK5/MEF2 axis^28,30,51,52^, but it is not yet known how radiation, particularly fractionated radiation, affects this axis. The current study revealed that fractionated radiation, compared to single exposure, significantly suppressed ERK5 phosphorylation and MEF2 expression, which potentially downregulate KLF2, TM, and eNOS and upregulate ICAM-1. Our results are consistent with the report of Deng *et al*. that showed ERK5-specific pharmacological inhibitors not only suppress KLF2 and its downstream targets but also enhance ICAM-1 expression^34^. These data clearly indicate that activation of the MEK5-ERK5 pathway may attenuate endothelial dysfunction induced by fractionated radiation.

Inhibitors of the mevalonate pathway activate the MEK5-ERK5 pathway^37,52^. The mevalonate metabolic pathway synthesizes three major types of cellular components: (1) sterol isoprenoids (e.g., cholesterol), (2) nonsterol isoprenoids (e.g., dolichol, heme-A, isopentenyl tRNA, ubiquinone), and (3) geranylgeranyl pyrophosphate (GGPP). GGPP donates a geranylgeranyl group for posttranslational modification of target proteins, a reaction catalyzed by geranylgeranyl transferase (GGT). This pathway converts HMG-CoA to mevalonate, and HMG-CoA reductase is the rate-limiting enzyme. Various groups have demonstrated that statins, competitive inhibitors of HMG-CoA reductase, upregulate KLF2 via ERK5 activation^52^. However, no study has investigated whether GT3, which can also inhibit HMG-CoA reductase by triggering posttranslational degradation of the enzyme, has efficacy similar to that of statins for ERK5 activation in endothelial cells. Notably, we demonstrated that GT3 and statins synergistically upregulate expression of KLF2 and TM and generation of APC in endothelial cells^38^, which are under positive control of ERK5 activation. In addition, GGTi upregulates KLF2 via ERK5 activation^37^. These data clearly indicate that inhibition of the mevalonate pathway can exert positive effects on the ERK5-KLF2 pathway, but no studies have investigated the ability of these inhibitors to reverse the suppression of KLF2 and its downstream target molecules that is induced by fractionated radiation. We demonstrated that treatment with atorvastatin, GT3, and GGTi significantly prevented fractionated radiation from suppressing levels of KLF2, TM, and eNOS in HUVECs. These may be relevant for developing strategies to mitigate radiotherapy-induced vascular damage.

In conclusion, we demonstrated, for the first time, that radiation-dose fractionation had differential effects on ERK5-KLF2 pathway. Compared to a single exposure of the same total dose, fractionated radiation more profoundly suppressed ERK5 phosphorylation; decreased expression of MEF2, KLF2, TM, and eNOS; decreased generation of APC; declined KLF2’s binding ability to TM and eNOS promoters; and upregulated ICAM-1 expression in HUVECs. We also observed fractionated and single-exposure radiation produced no changes in the DNA-binding ability of NF-κB family members in HUVECs, except p50, which indicates that effects of fractionated radiation on expression of ICAM-1 may not be NF-κB-dependent. In addition, we found that different inhibitors of the mevalonate pathway had the potential to attenuate fractionated-radiation–induced adverse effects on the ERK5-KLF2 pathway. Finally, our *in vivo* data revealed that fractionated thoracic radiation resulted in greater suppression of KLF2 and enhancement of ICAM-1 in the lung tissue samples than single exposure to the same total dose. These findings will provide crucial insights when selecting dose fractionation to minimize the short-term and long-term adverse side effects of radiotherapy. However, considering the heterogeneity of endothelial cells, further investigation is required to confirm these results in endothelial cells of different origins. In addition, how different radiation regimens under hypoxic condition, which is known to downregulate KLF2 via miR-200b^53^, alter KLF2-mediated axes require additional investigation.

## Supplementary information

Supplementary Information.

## References

[1] Galley HF, Webster NR (2004). Physiology of the endothelium. British journal of anaesthesia.

[2] Esmon CT (2012). Protein C anticoagulant system–anti-inflammatory effects. Seminars in immunopathology.

[3] Forstermann U, Munzel T (2006). Endothelial nitric oxide synthase in vascular disease: from marvel to menace. Circulation.

[4] Ito T, Maruyama I (2011). Thrombomodulin: protectorate God of the vasculature in thrombosis and inflammation. Journal of thrombosis and haemostasis: JTH.

[5] Martin FA, Murphy RP, Cummins PM (2013). Thrombomodulin and the vascular endothelium: insights into functional, regulatory, and therapeutic aspects. American journal of physiology. Heart and circulatory physiology.

[6] Tousoulis D, Kampoli AM, Tentolouris C, Papageorgiou N, Stefanadis C (2012). The role of nitric oxide on endothelial function. Current vascular pharmacology.

[7] Esmon CT (2003). The protein C pathway. Chest.

[8] Endemann DH, Schiffrin EL (2004). Endothelial dysfunction. Journal of the American Society of Nephrology: JASN.

[9] Schram MT, Stehouwer CD (2005). Endothelial dysfunction, cellular adhesion molecules and the metabolic syndrome. Hormone and metabolic research = Hormon- und Stoffwechselforschung = Hormones et metabolisme.

[10] Krieglstein CF, Granger DN (2001). Adhesion molecules and their role in vascular disease. American journal of hypertension.

[11] Collins T (1995). Transcriptional regulation of endothelial cell adhesion molecules: NF-kappa B and cytokine-inducible enhancers. FASEB journal: official publication of the Federation of American Societies for Experimental Biology.

[12] Ledebur HC, Parks TP (1995). Transcriptional regulation of the intercellular adhesion molecule-1 gene by inflammatory cytokines in human endothelial cells. Essential roles of a variant NF-kappa B site and p65 homodimers. The Journal of biological chemistry.

[13] Dumnicka, P. *et al*. The Interplay between Inflammation, Coagulation and Endothelial Injury in the Early Phase of Acute Pancreatitis: Clinical Implications. *International journal of molecular sciences* **18**, 10.3390/ijms18020354 (2017). 10.3390/ijms18020354 PMC5343889 28208708

[14] van Hinsbergh VW (2012). Endothelium–role in regulation of coagulation and inflammation. Seminars in immunopathology.

[15] Rajendran P (2013). The vascular endothelium and human diseases. International journal of biological sciences.

[16] Vanhoutte PM, Shimokawa H, Tang EH, Feletou M (2009). Endothelial dysfunction and vascular disease. Acta physiologica.

[17] Pathak R (2015). Thrombomodulin contributes to gamma tocotrienol-mediated lethality protection and hematopoietic cell recovery in irradiated mice. PloS one.

[18] Han, N. K. *et al*. Geranylgeranylacetone Ameliorates Intestinal Radiation Toxicity by Preventing Endothelial Cell Dysfunction. *International journal of molecular sciences* **18**, 10.3390/ijms18102103 (2017). 10.3390/ijms18102103 PMC5666785 28991157

[19] Geiger H (2012). Pharmacological targeting of the thrombomodulin-activated protein C pathway mitigates radiation toxicity. Nature medicine.

[20] Holler V (2009). Pravastatin limits radiation-induced vascular dysfunction in the skin. The Journal of investigative dermatology.

[21] Jeong YJ (2015). Coniferyl aldehyde attenuates radiation enteropathy by inhibiting cell death and promoting endothelial cell function. PloS one.

[22] Beckman JA, Thakore A, Kalinowski BH, Harris JR, Creager MA (2001). Radiation therapy impairs endothelium-dependent vasodilation in humans. Journal of the American College of Cardiology.

[23] Hauer-Jensen, M., Kong, F. M., Fink, L. M. & Anscher, M. S. Circulating thrombomodulin during radiation therapy of lung cancer. *Radiation oncology investigations* **7**, 238–242, 10.1002/(SICI)1520-6823(1999)7:4<238::AID-ROI5>3.0.CO;2-4 (1999). 10.1002/(SICI)1520-6823(1999)7:4<238::AID-ROI5>3.0.CO;2-4 10492164

[24] Wang J, Zheng H, Ou X, Fink LM, Hauer-Jensen M (2002). Deficiency of microvascular thrombomodulin and up-regulation of protease-activated receptor-1 in irradiated rat intestine: possible link between endothelial dysfunction and chronic radiation fibrosis. The American journal of pathology.

[25] Lin Z (2005). Kruppel-like factor 2 (KLF2) regulates endothelial thrombotic function. Circulation research.

[26] Lin Z (2010). Kruppel-like factor 2 regulates endothelial barrier function. Arteriosclerosis, thrombosis, and vascular biology.

[27] Pathak R (2014). IKKbeta regulates endothelial thrombomodulin in a Klf2-dependent manner. Journal of thrombosis and haemostasis: JTH.

[28] SenBanerjee S (2004). KLF2 Is a novel transcriptional regulator of endothelial proinflammatory activation. The Journal of experimental medicine.

[29] Wani MA, Wert SE, Lingrel JB (1999). Lung Kruppel-like factor, a zinc finger transcription factor, is essential for normal lung development. The Journal of biological chemistry.

[30] Kumar A, Lin Z, SenBanerjee S, Jain MK (2005). Tumor necrosis factor alpha-mediated reduction of KLF2 is due to inhibition of MEF2 by NF-kappaB and histone deacetylases. Molecular and cellular biology.

[31] Lee HY, Youn SW, Oh BH, Kim HS (2012). Kruppel-like factor 2 suppression by high glucose as a possible mechanism of diabetic vasculopathy. Korean circulation journal.

[32] Shi H (2013). Kruppel-like factor 2 protects against ischemic stroke by regulating endothelial blood brain barrier function. American journal of physiology. Heart and circulatory physiology.

[33] Hamik A (2007). Kruppel-like factor 4 regulates endothelial inflammation. The Journal of biological chemistry.

[34] Deng Y (2018). ERK5/KLF2 activation is involved in the reducing effects of puerarin on monocyte adhesion to endothelial cells and atherosclerotic lesion in apolipoprotein E-deficient mice. *Biochimica et biophysica acta*. Molecular basis of disease.

[35] Kim M (2012). Laminar flow activation of ERK5 protein in vascular endothelium leads to atheroprotective effect via NF-E2-related factor 2 (Nrf2) activation. The Journal of biological chemistry.

[36] Komaravolu RK (2015). Erk5 inhibits endothelial migration via KLF2-dependent down-regulation of PAK1. Cardiovascular research.

[37] Chu UB, Duellman T, Weaver SJ, Tao Y, Yang J (2015). Endothelial protective genes induced by statin are mimicked by ERK5 activation as triggered by a drug combination of FTI-277 and GGTI-298. Biochimica et biophysica acta.

[38] Pathak, R., Ghosh, S. P., Zhou, D. & Hauer-Jensen, M. The Vitamin E Analog Gamma-Tocotrienol (GT3) and Statins Synergistically Up-Regulate Endothelial Thrombomodulin (TM). *International journal of molecular sciences* **17**, 10.3390/ijms17111937 (2016). 10.3390/ijms17111937 PMC5133932 27869747

[39] Garg, S. *et al*. Gamma-Tocotrienol Protects the Intestine from Radiation Potentially by Accelerating Mesenchymal Immune Cell Recovery. *Antioxidants* **8**, 10.3390/antiox8030057 (2019). 10.3390/antiox8030057 PMC6466604 30845647

[40] Sangwung P (2017). KLF2 and KLF4 control endothelial identity and vascular integrity. JCI insight.

[41] Pathak R, Kumar VP, Hauer-Jensen M, Ghosh SP (2019). Enhanced Survival in Mice Exposed to Ionizing Radiation by Combination of Gamma-Tocotrienol and Simvastatin. Military medicine.

[42] Barnett GC (2009). Normal tissue reactions to radiotherapy: towards tailoring treatment dose by genotype. Nature reviews. Cancer.

[43] Kim JH, Jenrow KA, Brown SL (2014). Mechanisms of radiation-induced normal tissue toxicity and implications for future clinical trials. Radiation oncology journal.

[44] Rannou E (2015). *In vivo* evidence for an endothelium-dependent mechanism in radiation-induced normal tissue injury. Scientific reports.

[45] Wang J, Boerma M, Fu Q, Hauer-Jensen M (2007). Significance of endothelial dysfunction in the pathogenesis of early and delayed radiation enteropathy. World journal of gastroenterology.

[46] Richter KK (1998). Differential effect of radiation on endothelial cell function in rectal cancer and normal rectum. American journal of surgery.

[47] Wu X, Zhang JZ, Yang PF, Huang QH, Liu JM (2017). Regulation of Kruppel-like factor 2 (KLF2) in the pathogenesis of intracranial aneurysm induced by hemodynamics. American journal of translational research.

[48] Cervelli T (2014). Effects of single and fractionated low-dose irradiation on vascular endothelial cells. Atherosclerosis.

[49] Kim I (2001). Vascular endothelial growth factor expression of intercellular adhesion molecule 1 (ICAM-1), vascular cell adhesion molecule 1 (VCAM-1), and E-selectin through nuclear factor-kappa B activation in endothelial cells. The Journal of biological chemistry.

[50] Le NT (2014). Identification of activators of ERK5 transcriptional activity by high-throughput screening and the role of endothelial ERK5 in vasoprotective effects induced by statins and antimalarial agents. Journal of immunology.

[51] Clark PR (2011). MEK5 is activated by shear stress, activates ERK5 and induces KLF4 to modulate TNF responses in human dermal microvascular endothelial cells. Microcirculation.

[52] Wu K, Tian S, Zhou H, Wu Y (2013). Statins protect human endothelial cells from TNF-induced inflammation via ERK5 activation. Biochemical pharmacology.

[53] Bartoszewski R (2017). miR-200b downregulates Kruppel Like Factor 2 (KLF2) during acute hypoxia in human endothelial cells. European journal of cell biology.

