# Supplementary material for: Fractionated radiation suppresses Kruppel-like factor 2 pathway to a greater extent than by single exposure to the same total dose
Source: Sci Rep. 2020 May 7;10:7734. doi: 10.1038/s41598-020-64672-3 (PMC7206069; doi:10.1038/s41598-020-64672-3)
Supplement: Supplementary file 1 — Supplementary Information. [file 41598_2020_64672_MOESM1_ESM.pdf]

Title: Fractionated radiation suppresses Kruppel-like factor 2 pathway to a greater extent than by single exposure to the same total dose

Ratan Sadhukhan[1], Justin W. C. Leung[2], Sarthak Garg[1], Kimberly J. Krager1, Alena V. Savenka[3], Alexei G. Basnakian[3,4], Rupak Pathak[1*]

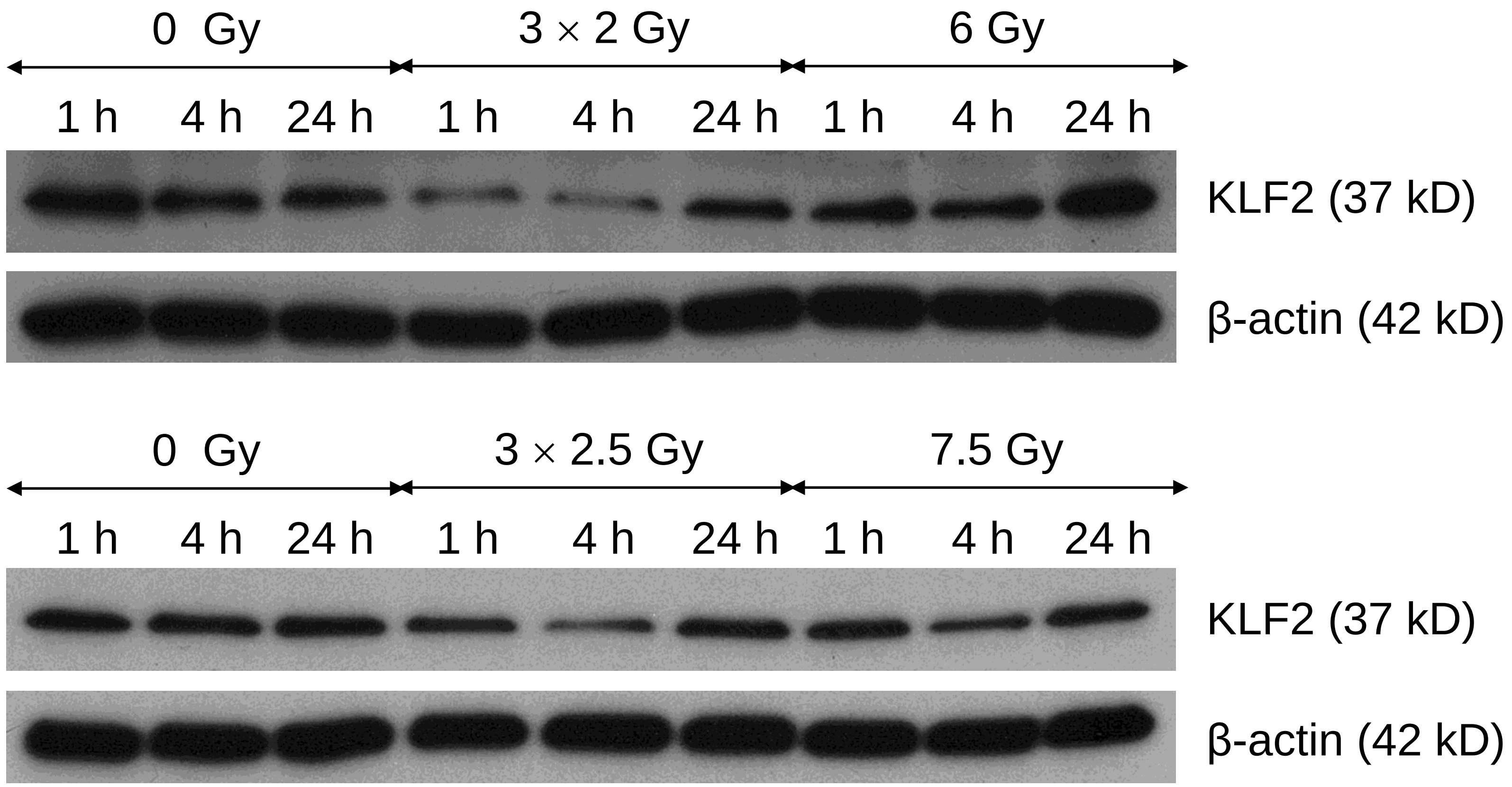

Suppl. Fig. 1: Fractionated radiation has more profound effect in suppressing KLF2 compared to single exposure. Representative Western blot analysis of KLF2 levels in un-irradiated (0 Gy) and irradiated HUVECs after 1 h, 4 h, and 24 h of exposure to 3 fractions of either 2 Gy or 2.5 Gy and a single exposure to either 6 Gy or 7.5 Gy, at 24 h inter-fraction intervals in whole-cell lysates from 2 independent experiments. β-actin serves as a loading control.

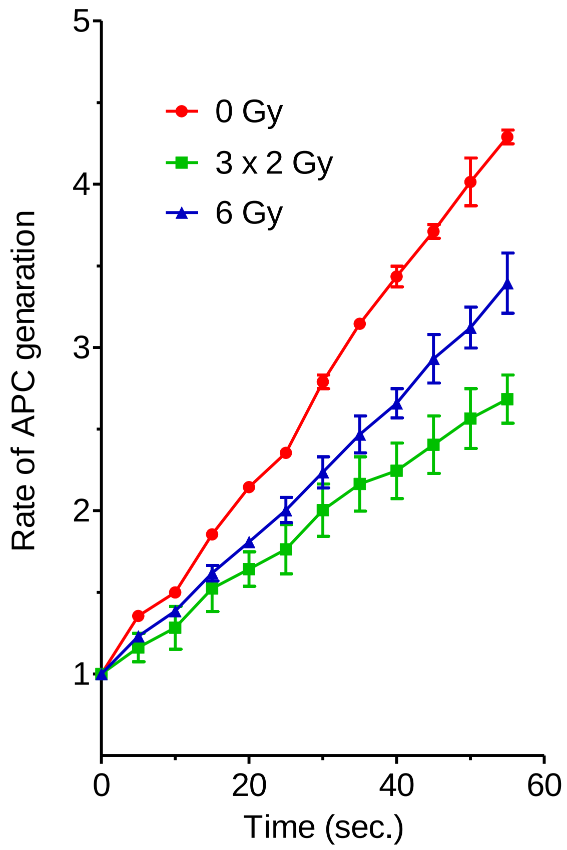

Suppl. Fig. 2: Fractionated radiation has more profound effect in suppressing APC generation compared to single exposure. APC generation in HUVECs after 24 h of exposure to 0 Gy (sham), 3 fractions of 2 Gy, and single exposure to 6 Gy. Experiments were performed twice (n = 2) with four biological replicates. “n” representing number of independent experiments performed.

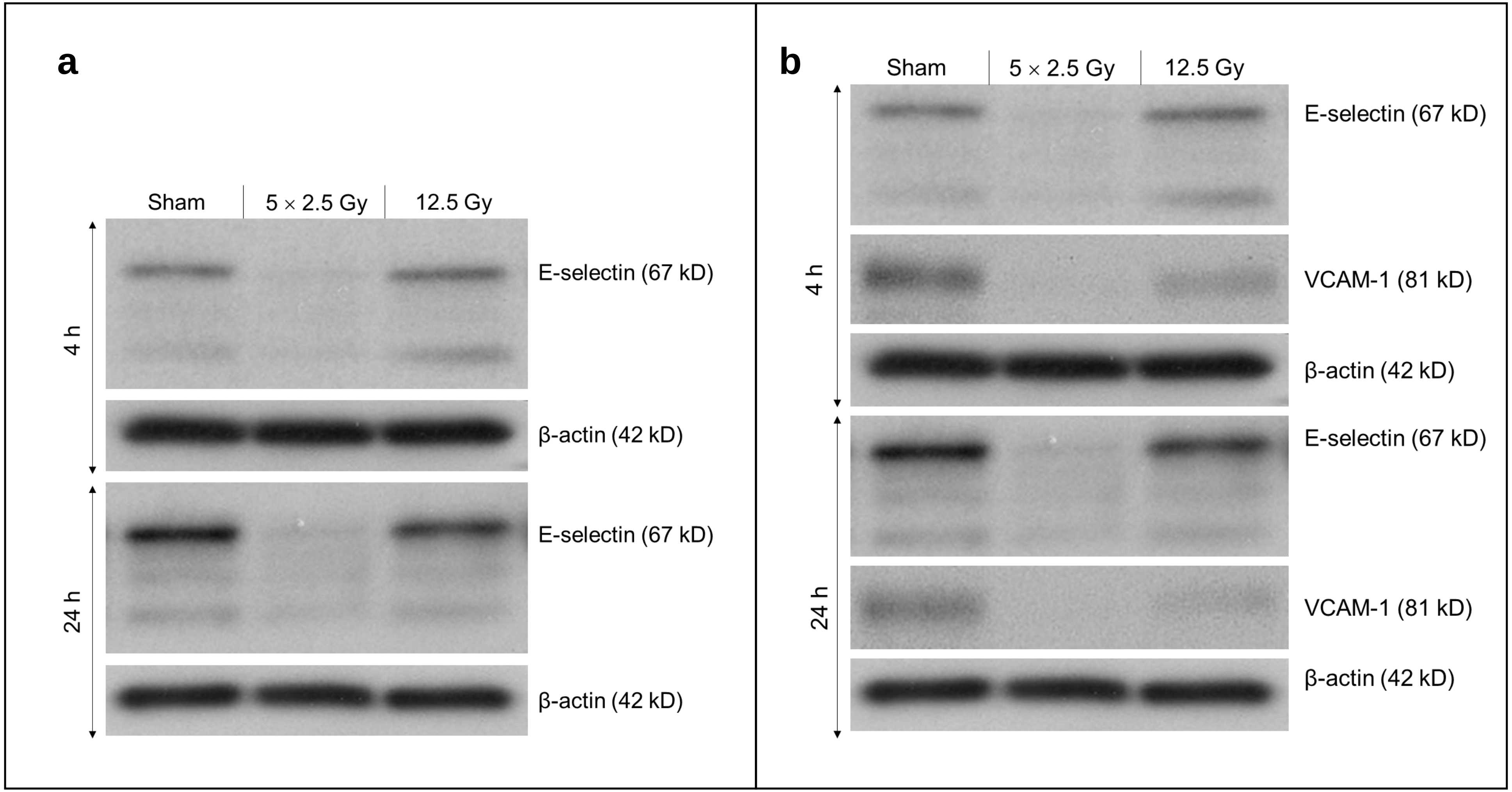

Suppl. Fig. 3: Fractionated radiation has more profound effect in suppressing E-selectin and VCAM-1 compared to single exposure. (**a**) Representative Western blot analysis of E-selectin levels in un-irradiated (0 Gy) and irradiated HUVECs after 4 h, and 24 h of exposure to 5 fractions of either 2 Gy and a single exposure to 10 Gy, at 24 h inter-fraction intervals in whole-cell lysates from 3 independent experiments. (**b**) Representative Western blot analysis of E-selectin and VCAM-1 levels in un-irradiated (0 Gy) and irradiated HUVECs after 4 h, and 24 h of exposure to 5 fractions of either 2.5 Gy and a single exposure to 12.5 Gy, at 24 h inter-fraction intervals in whole-cell lysates from 3 independent experiments β-actin serves as a loading control.
